# A pilot study using low-dose Spectral CT and ASIR (Adaptive Statistical Iterative Reconstruction) algorithm to diagnose solitary pulmonary nodules

**DOI:** 10.1186/s12880-015-0096-6

**Published:** 2015-11-17

**Authors:** Huijuan Xiao, Yihe Liu, Hongna Tan, Pan Liang, Bo Wang, Lei Su, Suya Wang, Jianbo Gao

**Affiliations:** The Department of Radiology, The First Affiliated Hospital of Zhengzhou University, No.1, East Jianshe Road, Zhengzhou, Henan Province 450052 China; The No.7 People’s Hospital of Zhengzhou, 17 Jingnan 5th Road, Zhengzhou Economic and Technological Development Zone, Zhengzhou, Henan Province 450000 China

**Keywords:** Computed tomography, Spectral CT, Solitary pulmonary nodules, Adaptive statistical iterative reconstruction

## Abstract

**Background:**

Lung cancer is the most common cancer which has the highest mortality rate. With the development of computed tomography (CT) techniques, the case detection rates of solitary pulmonary nodules (SPN) has constantly increased and the diagnosis accuracy of SPN has remained a hot topic in clinical and imaging diagnosis. The aim of this study was to evaluate the combination of low-dose spectral CT and ASIR (Adaptive Statistical Iterative Reconstruction) algorithm in the diagnosis of solitary pulmonary nodules (SPN).

**Methods:**

62 patients with SPN (42 cases of benign SPN and 20 cases of malignant SPN, pathology confirmed) were scanned by spectral CT with a dual-phase contrast-enhanced method. The iodine and water concentration (IC and WC) of the lesion and the artery in the image that had the same density were measured by the GSI (Gemstone Spectral Imaging) software. The normalized iodine and water concentration (NIC and NWC) of the lesion and the normalized iodine and water concentration difference (ICD and WCD) between the arterial and venous phases (AP and VP) were also calculated. The spectral HU (Hounsfield Unit ) curve was divided into 3 sections based on the energy (40–70, 70–100 and 100–140 *k*eV) and the slopes (λHU) in both phases were calculated. The IC_AP_, IC_VP_, WC_AP_ and WC_VP_, NIC and NWC, and the λHU in benign and malignant SPN were compared by independent sample *t-test*.

**Results:**

The iodine related parameters (IC_AP_, IC_VP_, NIC_AP_, NIC_VP_, and the ICD) of malignant SPN were significantly higher than that of benign SPN (*t* = 3.310, 1.330, 2.388, 1.669 and 3.251, respectively, *P* <0.05). The 3 λHU values of venous phase in malignant SPN were higher than that of benign SPN (*t* = 3.803, 2.846 and 3.205, *P* <0.05). The difference of water related parameters (WC_AP_, WC_VP_, NWC_AP_, NWC_VP_ and WCD) between malignant and benign SPN were not significant (*t* = 0.666, 0.257, 0.104, 0.550 and 0.585, *P* >0.05).

**Conclusions:**

The iodine related parameters and the slope of spectral curve are useful markers to distinguish the benign from the malignant lung diseases, and its application is extremely feasible in clinical applications.

## Background

Lung cancer is the most common cancer which has the highest mortality rate. In the past decades, the incidence of lung cancer has gradually increased in China [1, 2]. With the development of computed tomography (CT) techniques, the case detection rates of solitary pulmonary nodules (SPN) has constantly increased and the diagnosis accuracy of SPN has remained a hot topic in clinical and imaging diagnosis. Contrast-enhanced CT of the chest still remains the standard imaging test for the initial assessment of patients with suspected lung cancer. Using standard contrast-enhanced CT the characterization of pulmonary nodules is based on simple morphological criteria e.g., irregular or spiculated margins as a sign for malignancy or calcifications as a sign of benignity. However, in a clinical context these simple morphologic criteria are unreliable for an accurate differentiation between benign and malignant lung nodules.

X-ray computed tomography (CT) is a medical imaging modality that allows reconstruction of the internal stucture of the human body from a large number of x-ray attenuation measurements. The spectral CT in which the energy dependence of the x-ray attenuation coefficient is utilized Multiple parameters can be acquired by means of spectral CT techniques, such as monochromatic imaging, material decomposition images, spectral HU curve and effective atomic number, etc. [3, 4] ASIR (Adaptive Statistical Iterative Reconstruction) algorithm, is a compromise that relies on the accurate modeling of the noise distribution of the acquired data, rather than modeling the system optics. The result is an algorithm that is computationally fast and is effective at reducing noise, enabling radiation dose reductions that would not be possible [5–12]. The ASIR reconstruction algorithm is a promising technique for providing diagnostic quality CT images at significantly reduced radiation doses. ASIR is also helpful in improving CT image quality for obese patients.

In this study, patients with solitary pulmonary nodules (SPN) underwent dual-phase scanning by low dose spectral CT. The iodine and water concentrations were derived and the spectral HU curves were also acquired. By calculating and comparing the normalized concentration of iodine and water, the slopes of spectral curves in the benign and malignant SPN, the practical value of multiple parameters which was acquired by low dose spectral CT in SPN diagnosis are discussed. In this paper we propose an improved method to detect SPN. By analysis of different comparison parameters between benign and malignant pulmonary nodules and provide a reference for clinical diagnosis and treatment.

## Methods

### Design and setting

For this study, the use of medical imaging was approved by Medical Ethical Committee of The First Affiliated Hospital of Zhengzhou University. Approval was granted in accordance with Chinese legislations, and written informed consent was obtained from all participants, in accordance with the guidelines of the Chinese Ministry of Health. 64 patients with SPN received dual phase spectral CT scan between December 2013 and November 2014, but only 62 patients were included in the research. One case was excluded because the patient did not hold the breath and caused too many unacceptable motion artifacts; the other case was excluded since the solid lesion was too small to allow determination of the region of interest (ROI). The average age of 62 patients was 60 (ages from 42 to 80), including 40 males and 22 females. All the SPN cases were confirmed by surgery, trans-bronchial biopsy and pathology. Some patients with inflammatory nodules improved after anti-inflammatory therapy which was evident, clinically. There were totally 42 patients with malignant SPN (including 25 cases of adenocarcinoma; 13 cases of squamous carcinoma; 2 cases of bronchioloalveolar carcinoma; 1 case of mucoepidermoid carcinoma and 1 case of metastasis) and 20 patients with benign SPN (including 9 cases of inflammation; 7 cases of tuberculoma; 2 cases of hamartoma and 2 cases of sclerosing hemangioma). For use of these clinical materials for research purposes, prior consent from the patients and approval from the Research Ethics Committee of The First Affiliated Hospital of Zhengzhou University were obtained. All specimens were handled and made anonymous according to the ethical and legal standards.

### Diagnosis method

All patients underwent a two-phase contrast-enhanced low-dose spectral CT(GE Discovery CT750HD) examination with a single tube, and fast kilovoltage switching between 80 kVp and 140 kVp in less than 0.5 ms (GSI mode). Patients were examined 30 s (artery phase) and 60 s (venous phase) after contrast medium injection respectively. The scanning parameters were: 40 % ASIR (40 % ASIR images combined with 60 % FBP reconstruction image); tube current 260 mA; helical pitch 1.375:1; rotation speed 0.8 s; slice thickness 1.25 mm; detector coverage 40 mm, field-of-view (FOV) 32 cm, and CT dose index volume (CTDIVol) of 4.17 mGy per phase. Non-ionic contrast medium Iodixanol (Visipaque® 270 mg I/ml,, GE HealthCare) with antecubital venous access through power injector (Urich REF XD 2060-Touch, Germany) at a rate of 3–4 mL/s for a total of 90–120 mL (1.5 mL/kg, 80 ~ 100 ml).

### Image analysis

All the data were processed and analyzed by GSI Volume Viewer software package at AW4.6 work station (GE HealthCare, USA). The images were independently analyzed by two radiologists who had 5 and 10 years of experience, respectively. During the data analysis, the radiologists were able to adjust the window width and position based on the condition of each imaging. A circularregions of interest (ROI) was placed in the area that encompassed the entire tumor, as large as possible to reduce noise (.50 pixels), away from any peripheral fat and necrotic area. All measurements were repeated three times at the three contiguous imaging levels and average values were calculated to ensure consistency. In the iodine density image derived from the iodine/water based material decomposition image, the concentration of iodine (IC) and water (WC) in lesions (ICles and WCles) were measured in both arterial phase (AP) and venous phase (VP). In the same slice, the concentration of iodine and water in aorta descendens or subclavian artery (IC_ao_ and WC_ao_) were also measured. The normalized iodine concentration (NIC), which is the ratio of iodine concentration in lesion and aorta descendens (NIC = IC_les_/IC_ao_) and normalized water concentration (NWC, NWC = WC_les_/WC_ao_) were calculated. The iodine concentration difference (ICD, ICD = NIC_VP_-NIC_AP_, where the NIC_AP_ and NIC_VP_ are the normalized iodine concentration in arterial phase and venous phase, respectively) was calculated, and the water concentration difference (WCD, WCD = NWC_VP_-NWV_AP_) was calculated in the same manner. The spectral HU curve was divided into 3 regions, 40–70 *k*eV, 70–100 *k*eV and 100–140 *k*eV. The slope (λHU) of 40–70 keV was calculated by the equations K40-70 keV = (40 keV-70 keV) HU/70-40 which was the same with The slope (λHU) of 70–100 keV and 100–140 keV.

### Statistical analysis

All data was analyzed by SPSS 17.0 software package. The measurement data was displayed as s ± d (mean ± deviation), and independent-samples *t*-test was used in the differential analysis (α = 0.05). The results were considered statistically significant when *P* < 0.05.

## Results

### Comparison of IC, NIC and ICD in both benign and malignant SPN

In malignant SPN, the IC in both arterial and venous phase, NIC and ICD were significantly higher than benign SPN when statistically analyzed. The results are shown in Table 1.

**Table 1:** The comparison of IC, NIC and ICD in benign and malignant SPN

	Malignant SPN (*n* = 42)	Benign SPN (*n* = 20)	*t* value	*P* value
IC_AP_	19.322 ± 5.554	11.711 ± 3.724	3.310	0.003
IC_VP_	19.191 ± 6.438	15.297 ± 6.258	1.330	0.014
NIC_AP_	0.163 ± 0.056	0.112 ± 0.028	2.388	0.027
NIC_VP_	0.649 ± 0.888	0.286 ± 0.078	1.669	0.035
ICD	0.264 ± 0.120	0.163 ± 0.061	3.251	0.002

### Comparison of WC, NWC and WCD in both benign and malignant SPN

WC in both arterial and venous phase, the NWC and WCD in malignant SPN have no statistically significant differences compared to benign SPN. The results are shown in Table 2.

**Table 2:** The comparison of WC, NWC and WCD in benign and malignant SPN

	Malignant SPN (*n* = 42)	Benign SPN (*n* = 20)	*t* value	*P* value
WC_AP_	1019.55 ± 14.407	1015.85 ± 13.438	0.666	0.511
WC_VP_	1020.61 ± 12.598	1018.84 ± 19.568	0.257	0.801
NWC_AP_	1.014 ± 0.021	1.015 ± 0.019	0.104	0.918
NWC_VP_	1.005 ± 0.021	1.001 ± 0.017	0.550	0.587
WCD	0.008 ± 0.022	0.013 ± 0.021	0.585	0.563

### Calculation and comparison of spectral curve slope (λHU) at arterial and venous phase in benign and malignant SPN

**Table 3:** The slope of 3 energy decay curve sections in benign and malignant SPN

Groups	Arterial phase			Venous phase		
	40-70 *k*eV	70-100 *k*eV	100-140 *k*eV	40-70 *k*eV	70-100 *k*eV	100-140 *k*eV
Malignant SPN(*n* = 42)	3.473 ± 1.121	0.781 ± 2.975	0.359 ± 0.119	4.147 ± 1.356	1.793 ± 1.465	0.425 ± 0.141
Benign SPN(*n* = 20)	3.396 ± 2.578	0.835 ± 0.712	0.357 ± 0.276	2.670 ± 0.697	0.761 ± 0.351	0.289 ± 0.083
*t* value	0.09	0.231	0.030	3.803	2.846	3.205
*P* value	0.930	0.822	0.976	0.001	0.010	0.004

**Fig. 1:**
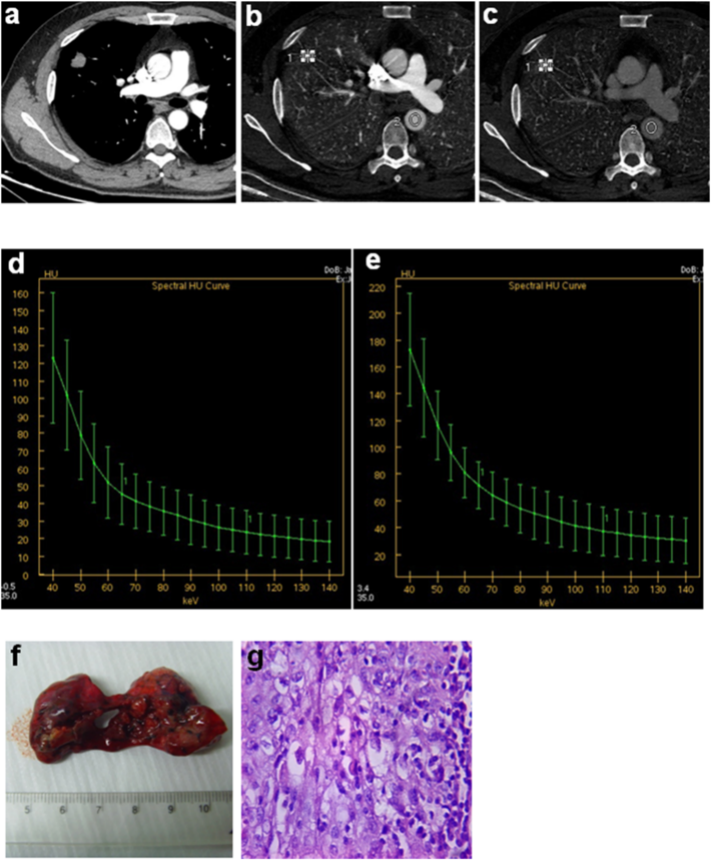
Male, 47 years old. Space occupying lesion was found in the right upper lobe. Middle differentiation squamous cell carcinomas, confirmed by the postoperative pathological diagnosis. **a** monochromatic image, 70 *k*eV, arterial and venous phase; **b** arterial phase iodine image, IC_AP_ = 13.28 mg/ml; **c** venous phase iodine image, IC_VP_ = 18.1 mg/ml; **d** arterial phase spectral energy curve; **e** venous phase spectral energy curve, the slope of 40–70, 70–100 and 100–140 *k*eV are 3.72, 0.66, 0.36, respectively; **f** pathologic samples after surgery; **g** pathological section image (HE dye, ×400)

**Fig. 2:**
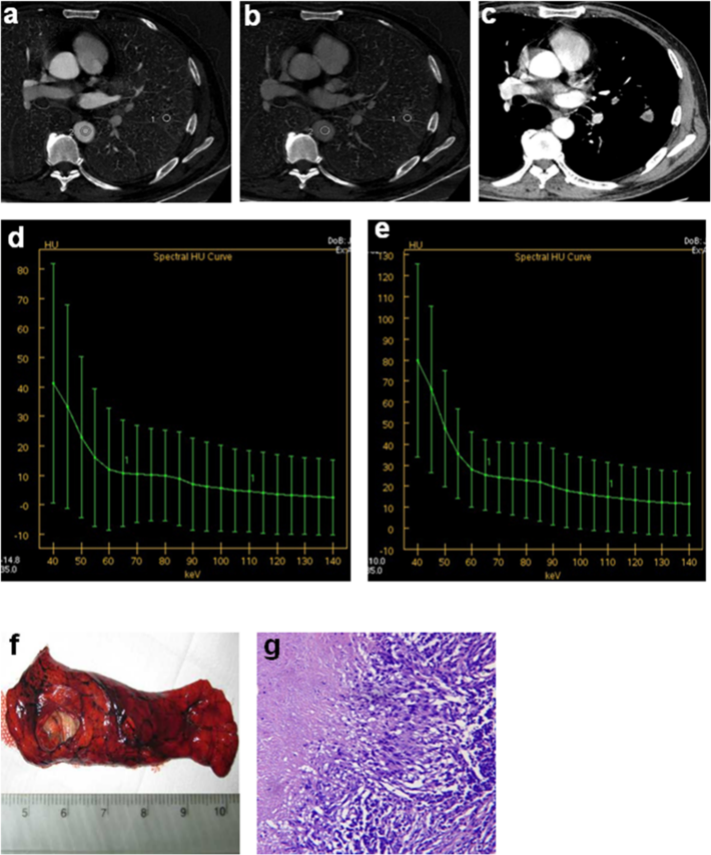
Male, 57 years old. Pulmonary nodule was found in left upper lobe. Tuberculosis in lower left lobe confirmed by postoperative pathological diagnosis **a** monochromatic image, 70 *k*eV, arterial and venous phase; **b** arterial phase iodine image, IC_AP_ = 8.43 mg/ml; **c** venous phase iodine image, IC_VP_ = 8.55 mg/ml; **d** arterial phase spectral energy curve; **e** venous phase spectral energy curve, the slope of 40–70, 70–100 and 100–140 *k*eV are 1.84, 0.25, 0.17, respectively; **f** pathologic samples after surgery; **g** pathological section image (HE dye, ×100)

The results showed that with the increase in *k*eV, the λHU in both benign and malignant SPN decreased, and the λHU of malignant SPN was larger than that of benign SPN, but the differences were reduced when *k*eV increased (Fig. 1 and Fig. 2). In the arterial phase, the 3 slopes of malignant and benign SPN have no significant differences (*P* >0.05); while in the venous phase, the 3 slopes of malignant SPN were significantly larger than that of benign SPN (*P* <0.05).So we can use the λHU of venous phase in lower keV to identify benign with malignant nodule. The results are shown in Table 3.

## Discussion

The methodology to diagnose SPN has been developing over the years from a traditional morphological examination to a dynamic functional examination. Spectral CT imaging has the potential to provide multiple techniques, such as polychromatic and monochromatic imaging, material decomposition imaging, etc. Therefore, spectral CT has been widely used in the detection of early stage cancer, qualitative diagnosis of neoplastic disease, staging diagnosis and reducing the metal artifacts and so on [13–17].

It was reported that tumor cells can produce angiogenic factors that stimulate and generate a large number of new blood vessels. Since the wall of these new blood vessels are immature there is a lack of hemangiopericyte and smooth muscle tissue and there is increased permeability of the blood vessels [18–20]. Lung cancer cells by themselves are highly aggressive; they can invade the corresponding artery making the terminal blood vessels get thicker and circuitous. Since there is a lack of venous and lymphatic drainage system in the cancer lesion, the contrast agent gets diffused into the extravascular space. But in the case of benign SPN, most of them do not have abundant blood supply and the vascular basement membrane is not damaged, so the permeability of blood vessels is not increased. Tateishi et al. analyzed the correlation of tumor enhancement with MVD and VEGF expression in 130 patients with histological proven lung cancer [21]. They found a significantly higher peak enhancement of VEGF-positive tumors than in VEGF-negative tumors. In this study, the ICAP, ICVP, NIC and ICD in malignant SPN were all significantly higher than that of benign SPN. In benign SPN, the active inflammatory nodule also contains more blood vessels due to the stimulation of inflammatory substances. With the conversion of an active nodule to a chronic inflammatory nodule, the fabric content in the lesion increases while the vascular content decreases. As a consequence, the iodine concentration in active inflammatory nodule may also be high, and comparable to a malignant nodule. In the chronic inflammatory and other benign nodule, due to the lack of blood vessel, the contrast agent diffuse slowly, therefore, in some case, the ICVP is higher than ICAP. It is similar to the study of Schmid-Bindert et al., who investigated the correlation between maximum standardized uptake value (SUV(max)) of (18) FDG PET-CT and iodine-related attenuation (IRA) of dual energy CT (DECT) of primary tumours and (18) FDG PET-CT positive thoracic lymph nodes (LN) in patients with lung cancer and a moderate correlation was found between SUV(max) and maximum IRA in all tumours [22].

In the iodine/water based material decomposition image, the water content was measured. Based on the calculation and statistical analysis, the results showed that WC, NWC and WCD in both benign and malignant SPN have no significant differences. In this study, the inflammatory nodule cases were relatively high in the benign SPN group. Since the blood flow of inflammatory nodule increases in the congestive stage, the water content in both intra- and extra-cellular is high. As for the malignant SPN, the water content in intra- and extra-cellular is also high due to the relatively higher vascular capacitance and large amount of tumor cells. The center of tuberculoma consists of caseous necrotic tissue surrounded by fibrous tissue, and the caseous necrotic tissue is a pink amorphous granular mass, which exhibits more severe necrosis: coagulative necrosis. Coagulative necrosis is enriched with lipid and less water content. In this study, the water content is high in inflammatory nodule cases, which led to the premise that water concentration related parameters (WC, NWC, WCD) are not significantly different between benign and malignant groups.

The iodine concentration in the ROI directly reflects the blood supply situation in the nodule. Research showed that the IC in malignant nodules is higher than in benign nodules [23]. Several studies have compared the CT numbers of pulmonary nodules on iodine-enhanced image with that on enhanced weighted average images The results of both the CT number on iodine-enhanced images and the degree of enhancement showed that malignant nodules showed a significantly higher enhancement than benign nodules (*P* = 0.001), and iodine-enhanced images had a higher sensitivity and accuracy than the degree of enhancement [24–28]. The spectra curve is a reflection of different lesions and different tissues or organs in the human body absorb X-rays at different rates. It shows the variation of CT values in different regions of different keV [29, 30].The difference of spectral curve needs to be correlated to the iodine concentration in the lesions when the contrast agent is applied. In this study, the slopes of spectral HU curve were decreased with the increase in keV in both benign and malignant nodules. However in all the 3 curve sections, the slopes of malignant SPN are all higher than the corresponding slopes of benign SPN.

The low dose spectral scan mode was applied in this research. The tube current was set to 260 mA, CTDIVol was 4.17 mGy per phase, which is significantly lower compared to the dose of the first generation technology. In addition, the ASIR algorithm reduced the noise and improved the imaging quality, and makes it possible to acquire good quality image in much lower dosage. The percentage of ASIR (10–100 %) is operator selectable at the console. It reflects a linear combination of the original FBP image (0 % ASIR) and an essentially noise-free image created by full compliance with the mathematic model (100 % ASIR). A choice of 40 % ASIR implies that 40 % of the ASIR image was blended with the FBP image. A preliminary phantom analysis and clinical feasibility study of low-dose body CT using 40 % ASIR determined that it provided quantitative and qualitative image noise and quality nearly identical to those of routine-dose CT [31].

In this study, the ultralow concentration iso-osmolar contrast agent (270mgI/mL) was given to the patients; consequently, the incidence of adverse reaction was reduced, which is a better choice of contrast agent when the patients have potential renal damage or cardiac insufficiency.

Although the results in this research are concrete and convincing, this work still has several aspects that need to be explored thoroughly in further studies, such as, expanding the cases; detailed study based on different pathological types of lung cancer; distinguishing the active inflammatory nodules from malignant nodules; comparing the water content related parameters in different pathological nodules, etc. In conclusion, the combination of low dose spectral CT and ASIR algorithm can help acquire multiple parameters under the low dose mode and acquiring these parameters is highly practicable in the clinic during diagnosis of benign and malignant SPN.

## Abbreviations

CT: computed tomography
ASIR: adaptive statistical iterative reconstruction
FBP: filtered back projection
SPN: solitary plmonary nodules
IC: iodine concentration
WC: water concentration
GSI: gemstone spectral imaging
NIC: normalized iodine concentration
NWC: normalized water concentration
ICD: iodine concentration difference
WCD: water concentration difference
AP: arterial phases
VP: venous phases
HU: Hounsfield Units
